# Long-Term Extensive Ectopic Hair Growth on the Spinal Cord of Mice from Transplanted Whisker Follicles

**DOI:** 10.1371/journal.pone.0133475

**Published:** 2015-08-05

**Authors:** Wenluo Cao, Lingna Li, Sumiyuki Mii, Yasuyuki Amoh, Fang Liu, Robert M. Hoffman

**Affiliations:** 1 AntiCancer Inc., San Diego, CA, United States of America; 2 Department of Surgery, University of California San Diego, San Diego, CA, United States of America; 3 Department of Anatomy, Second Military Medical University, Shanghai, China; 4 Department of Dermatology, Kitasato University, Sagimahara, Japan; National Cancer Institute, UNITED STATES

## Abstract

We have previously demonstrated that hair follicles contain nestin-expressing pluripotent stem cells that can effect nerve and spinal cord repair upon transplantation. In the present study, isolated whisker follicles from nestin-driven green fluorescent protein (ND-GFP) mice were histocultured on Gelfoam for 3 weeks for the purpose of transplantation to the spinal cord to heal an induced injury. The hair shaft was cut off from Gelfoam-histocultured whisker follicles, and the remaining part of the whisker follicles containing GFP-nestin expressing pluripotent stem cells were transplanted into the injured spinal cord of nude mice, along with the Gelfoam. After 90 days, the mice were sacrificed and the spinal cord lesion was observed to have healed. ND-GFP expression was intense at the healed area of the spinal cord, as observed by fluorescence microscopy, demonstrating that the hair follicle stem cells were involved in healing the spinal cord. Unexpectedly, the transplanted whisker follicles sprouted out remarkably long hair shafts in the spinal cord during the 90 days after transplantation of Gelfoam whisker histocultures to the injured spine. The pigmented hair fibers, grown from the transplanted whisker histocultures, curved and enclosed the spinal cord. The unanticipated results demonstrate the great potential of hair growth after transplantation of Gelfoam hair follicle histocultures, even at an ectopic site.

## Introduction

We previously discovered nestin-expressing cells, in the permanent upper hair follicle immediately below the sebaceous glands in the hair follicle bulge area, in nestin-driven green fluorescent protein (GFP) (ND-GFP) transgenic mice. The ND-GFP-expressing cells in the bulge area surrounded the hair shaft and were interconnected by short dendrites [1].

We subsequently demonstrated that ND-GFP stem cells isolated from the hair-follicle bulge area could differentiate into neurons, glia, keratinocytes, smooth muscle cells, and melanocytes *in vitro* [2].

Hair follicle stem cells from ND-GFP mice were transplanted into the gap region of severed sciatic nerves of nude mice. The transplanted stem cells enhanced the rate of nerve regeneration and the restoration of nerve function. The ND-GFP cells differentiated mostly into Schwann (glial) cells, which supported neuron regrowth [3].

Nestin-expressing hair follicle stem cells were subsequently transplanted to the injured spinal cord of nude mice. Most of the transplanted cells also differentiated into Schwann cells which facilitated repair of the severed spinal cord. The rejoined spinal cord resulted in extensive hind-limb locomotor performance recovery [4].

To understand the role of the ND-GFP stem cells within the hair follicle, whiskers from ND-GFP mice were placed in 3D Gelfoam histoculture where β-III tubulin-positive fibers, consisting of ND-GFP-expressing cells, extended up to 500 mm from the whisker nerve stump in Gelfoam histoculture. These fibers had growth cones on their tips expressing F-actin indicating that β-III tubulin-positive fibers, elongating from the whisker follicle sensory nerve stump, were axons. The elongated whisker sensory nerve was highly enriched in ND-GFP-cells [5].

ND-GFP-expressing BA and dermal papilla (DP) cells were histocultured on Gelfoam and were separately transplanted to the injured spinal cord of nude mice. Both DP and BA ND-GFP cells differentiated into neuronal and glial cells after transplantation to the injured spinal cord. ND-GFP cells from both areas enhanced injury repair and locomotor recovery [6, 7].

In the present study, we demonstrate the surprising result that Gelfoam-histocultured whisker follicles, transplanted to the injured spine, along with the Gelfoam on which they were histocultured, sprouted long hair shafts from the spinal cord.

## Materials and Methods

### Ethics Statement

All animal studies were conducted with an AntiCancer, Inc., Institutional Animal Care and Use Committee (IACUC)-protocol specifically approved for this study and in accordance with the principals and procedures outlined in the National Institute of Health Guide for the Care and Use of Animals under Assurance Number A3873-1. In order to minimize any suffering of the animals the use of anesthesia and analgesics were used for all surgical experiments. Animals were anesthetized by intramuscular injection of a 0.02 ml solution of 20 mg/kg ketamine, 15.2 mg/kg xylazine, and 0.48 mg/kg acepromazine maleate. The response of animals during surgery was monitored to ensure adequate depth of anesthesia. Ibuprofen (7.5 mg/kg orally in drinking water every 24 hours for 7 days post-surgery) was used in order to provide analgesia post-operatively in the surgically-treated animals. The animals were observed on a daily basis and humanely sacrificed by CO_2_ inhalation when they met the following humane endpoint criteria: prostration, skin lesions, significant body weight loss, difficulty breathing, epistaxis, rotational motion and body temperature drop. The use of animals was necessary to understand the role of transplanted pluripotent-stem-cell-containing hair follicles in the repair of injured mouse spinal cord and the ability of the hair follicles to produce hair shafts at the ectopic site. Animals were housed with no more than 5 per cage. Animals were housed in a barrier facility on a high efficiency particulate air (HEPA)-filtered rack under standard conditions of 12-hour light/dark cycles. The animals were fed an autoclaved laboratory rodent diet (S1 ARRIVE checklist).

### Mice

ND-GFP transgenic mice and non-transgenic nude mice (AntiCancer, Inc., San Diego, CA), at different ages, were used [6]. Mice were kept in a barrier facility under HEPA filtration (as noted above). Mice were fed with an autoclaved laboratory rodent diet. All mouse surgical procedures and imaging were performed with the animals anesthetized by subcutaneous injection of the ketamine mixture, described above. All animal studies were conducted with an AntiCancer Institutional Animal Care and Use Committee (IACUC)-protocol specifically approved for this study and in accordance with the principals and procedures outlined in the National Institute of Health Guide for the Care and Use of Animals under Assurance Number A3873-1, as described above.

### Isolation of mouse vibrissa follicles

The whisker pad of the nestin-GFP transgenic mice was removed and its inner surface was exposed and dissected under a binocular microscope with small scissors to separate each follicle. Intact follicles were chosen for Gelfoam histoculture. The isolated vibrissae were washed in PBS three times before Gelfoam histoculture (Pharmacia and Upjohn Co., Kalamazoo, MI) in DMEM-F12 medium (GIBCO Life Technologies, New. York, NY) containing B-27 (2.5%) (GIBCO Life Technologies), N2 (1%) (GIBCO Life Technologies), and 1% penicillin and streptomycin (GIBCO Life Technologies) [5].

### Surgical procedure for spinal cord injury and transplantation of Gelfoam whisker histoculture

Non-transgenic nude mice were anesthetized with a ketamine mixture (described above). The skin overlaying the vertebral column was cut. Fine scissors was used to separate the muscles along the spine and then a partial laminectomy was made at T8 with a 31G insulin syringe needle at the right side of the spinal cord. The lesion surface size was 500 μm × 500 μm and depth of the lesion was from the dorsal to the abdominal surface of the spinal cord. Gelfoam whisker cultures (including the Gelfoam) were introduced into the lesion site with fine forceps within 30 minutes of injury. There were no mortalities associated with this procedure. Fluorescence and bright light microscopy of the spinal cord was the endpoint. Animals were euthanized at day-90, at which point there were no mortalities.

### Statistical analyses

All data are presented as mean ± SEM. Group differences were obtained using the Fisher exact test. The significance level for all tests was set at p ≤ 0.05.

## Results and Discussion

### Ectopic growth of hair in the spinal cord after transplantation of whisker Gelfoam histoculture

**Fig 1:**
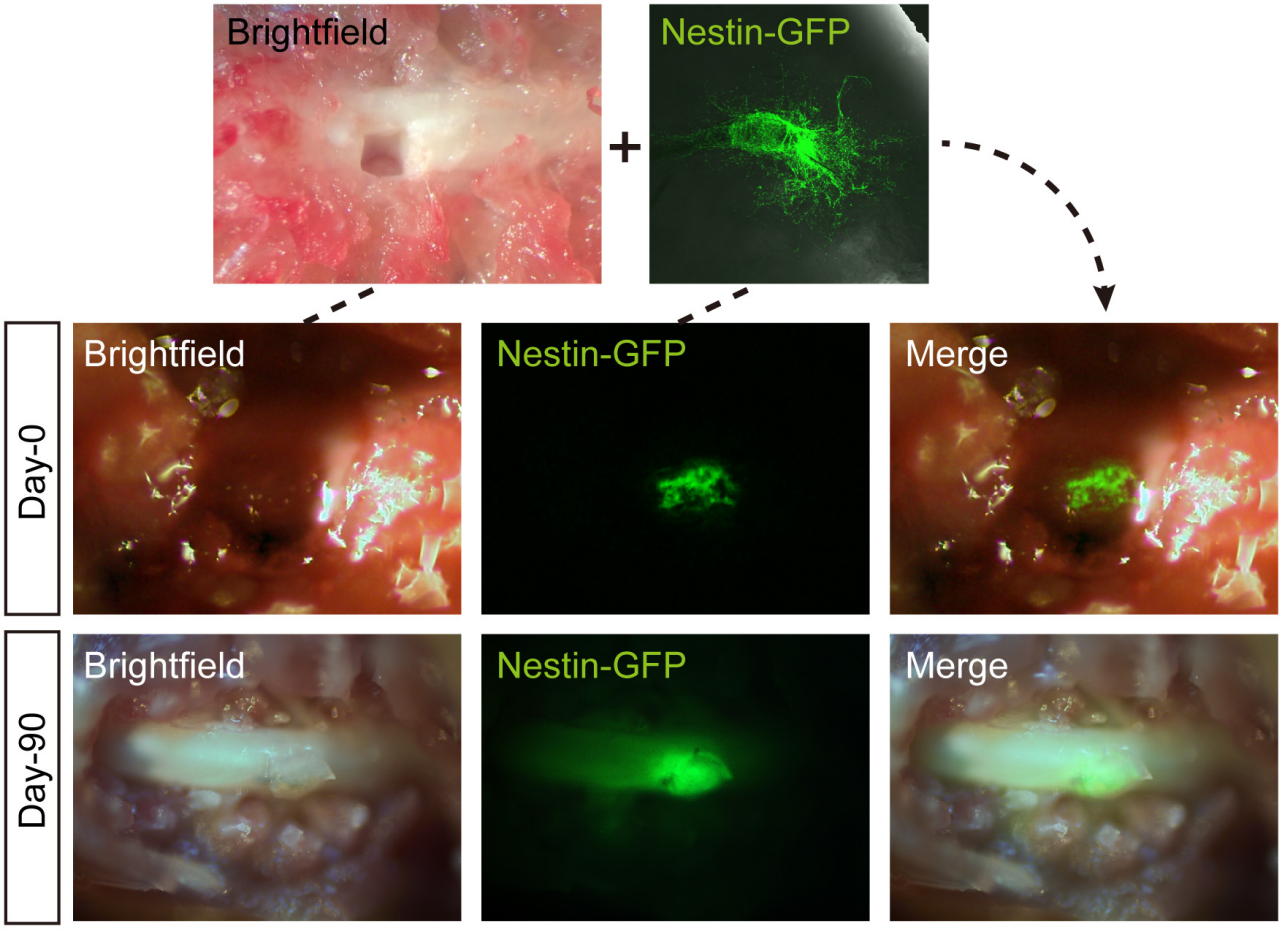
Transplantation of nestin-driven green fluorescent protein (ND-GFP)-expressing hair follicle Gelfoam histocultures to the injured spinal cord of nude mice. After Gelfoam histoculture of isolated whisker hair follicles from nestin-driven GFP (ND-GFP) mice for 3 weeks, the long hair shafts of the whisker follicle were cut off, and the follicle, along with the Gelfoam, was transplanted into the injured nude-mouse spinal cord. The transplanted mouse was sacrificed after 90 days. ND-GFP expression intensified by 90 days and expanded in the injured area of the spinal cord, which was apparently healed by the ND-GFP expressing stem cells. A total of 7 mice were studied. The figure shows typical data.

After Gelfoam histoculture of whiskers for 3 weeks, elongated hair shafts were cut off and the whisker follicles, containing ND-GFP stem cells, which had increased during histoculture, were transplanted along with the Gelfoam into the injured spinal cord of nude mice (Fig 1).

**Fig 2:**
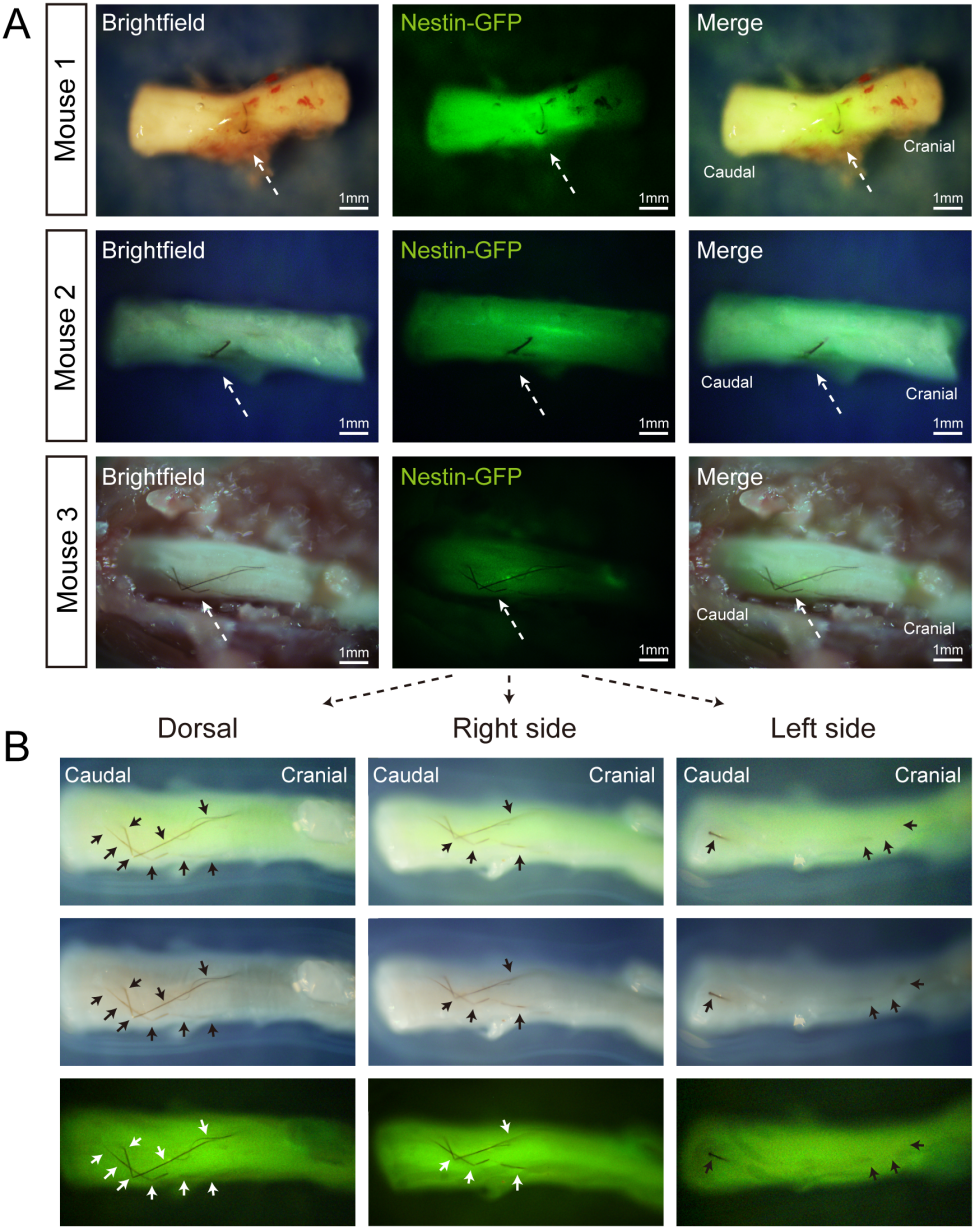
Ectopic hair growth in the spinal cord. Ninety days after transplantation of the 3-week Gelfoam ND-GFP-expressing whisker histocultures in the injured spinal cord, long hair shafts (arrows), were observed along and around the healed spinal cord. (**A**) Shows the elongated hair shafts that grew from whisker follicles, previously histocultured on Gelfoam into the injured spinal cord in 3 different mice at day-90 after surgery. All mice demonstrated hair shaft growth from the transplanted histoculture whisker follicles. Mouse 3 had the most remarkable hair shaft growth, which curved and enclosed the spinal cord. Arrows showed the hair growth in the spinal cord. (**B**) Panels show the hair shaft growth from the transplanted Gelfoam histoculture whisker follicles in the spine from mouse 3 at higher magnification from different views of the spinal cord (dorsal, left, and right side). The growing hair shaft reached a length of almost 14 mm and curved around the spinal cord. Arrows depict the hair shaft growing from the whisker hair follicles transplanted in the spine. Six out of 7 mice implanted with the Gelfoam whisker histoculture showed extensive ectopic hair growth on the spine.

After 90 days, the mice were sacrificed. At this time, the spinal-cord lesion appeared healed. ND-GFP expression was visible and intense along the healed area of the spinal cord, suggesting the hair follicle stem cells were viable and healed the injury. We previously reported that implantation of Gelfoam-supported whisker histocultures to the injured spinal cord resulted in functional healing. In the present experiment, the whiskers were histocultured for a longer period of time and the mice had a longer time after implantion before examination of their spinal cord. It was assumed that the spinal cord was functionally healed, as in our previous experiment [4, 6]. Unexpectedly, stout pigmented hair fibers were observed growing from the implanted hair follicle Gelfoam complex transplanted to the spinal cord (Fig 2). The hair shafts grew remarkably long in the spinal cord, as much as approximately 14 mm, and curved and enclosed the spinal cord (Fig 2, Mouse-3). A total of 7 mice were implanted with Gelfoam whisker histoculture and after examination, six mice showed ectopic hair growth (*p* = 0.001 compared to mice implanted with Gelfoam only).

Gelfoam, which is derived from gelatinized pig skin, provides a 3-dimensional physiological scaffold for the hair follicle to attach and grow, both in vitro and in the injured spinal cord, which may provide nutritional factors for long-term hair shaft growth [5, 6, 8, 9]. Gelfoam appears to preserve the integral hair follicle both in vitro and in vivo. The unanticipated results demonstrate the great potential of hair shaft growth after Gelfoam histoculture and transplantation of the hair follicle *in vivo*, even at an ectopic site. The length of the hair shaft observed in mouse 3 of almost 14 mm suggests that the ectopic site of the spine can strongly stimulate hair growth.

Future experiments will correlate kinetics of ectopic hair growth on the spine and the kinetics of spinal cord healing.

## Conclusions

The experimental system described in the present report can be used for further studies of healing spinal cord injury using transplanted hair follicle histocultures and also to study the conditions at this ectopic site promotes hair growth and to correlate the two.

## Supporting Information

S1 ARRIVE Checklist (PDF)
